# Inhibition of c-Kit Is Not Required for Reversal of Hyperglycemia by Imatinib in NOD Mice

**DOI:** 10.1371/journal.pone.0084900

**Published:** 2014-01-15

**Authors:** Janet Lau, Qiang Zhou, Susan E. Sutton, Ann E. Herman, Christian Schmedt, Richard Glynne

**Affiliations:** 1 JDRF Pharmacology, Genomics Institute of the Novartis Research Foundation, San Diego, California, United States of America; 2 Genetics and Neglected Diseases, Genomics Institute of the Novartis Research Foundation, San Diego, California, United States of America; 3 Immunology Discovery, Genomics Institute of the Novartis Research Foundation, San Diego, California, United States of America; Children's Hospital Boston, United States of America

## Abstract

**(1) Aim/Hypothesis:**

Recent studies indicate that tyrosine kinase inhibitors, including imatinib, can reverse hyperglycemia in non-obese diabetic (NOD) mice, a model of type 1 diabetes (T1D). Imatinib inhibits c-Abl, c-Kit, and PDGFRs. Next-generation tyrosine kinase inhibitors for T1D treatment should maintain activities required for efficacy while sparing inhibition of targets that might otherwise lead to adverse events. In this study, we investigated the contribution of c-Kit inhibition by imatinib in reversal of hyperglycemia in NOD mice.

**(2) Methods:**

The T670I mutation in c-Kit, which confers imatinib resistance, was engineered into the mouse genome and bred onto the NOD background. Hematopoietic stem cells (HSCs) from NOD.c-Kit^T670I^ mice and NOD.c-Kit^wt^ littermates were expanded in the presence or absence of imatinib to verify imatinib resistance of the c-Kit^T670I^ allele. Diabetic mice were treated with imatinib at the onset of hyperglycemia for three weeks, and blood glucose was monitored.

**(3 )Results:**

*In vitro* expansion of HSCs from NOD.c-Kit^wt^ mice was sensitive to imatinib, while expansion of HSCs from NOD.c-Kit^T670I^ mice was insensitive to imatinib. However, *in vivo* treatment with imatinib lowered blood glucose levels in both strains of mice.

**(4) Conclusions/Interpretation:**

The HSC experiment confirmed that, in NOD.c-Kit^T670I^ mice, c-Kit is resistant to imatinib. As both NOD.c-Kit^T670I^ and NOD.c-Kit^wt^ mice responded comparably to imatinib, c-Kit inhibition does not substantially contribute to the efficacy of imatinib in T1D. Thus, we conclude that inhibition of c-Kit is not required in next-generation tyrosine kinase inhibitors for T1D treatment, and may be selected against to improve the safety profile.

## Introduction

Type 1 Diabetes (T1D) is an autoimmune disease in which immune cells specifically target and kill pancreatic beta cells. A disease that typically manifests in young children, T1D necessitates a lifelong dependence on insulin, and is accompanied by significantly increased health risks and complications, even under the best managed care. Importantly, T1D affects an estimated 11–22 million people globally and its prevalence is increasing [1], [2]. This underscores the urgent need to find and develop treatments that can better regulate glycemia, restore beta cell function and improve patient outcomes.

Imatinib is a tyrosine kinase inhibitor (TKI) that was initially developed as an inhibitor of the Bcr-Abl oncogene for the treatment of chronic myeloid leukemia (CML) [3]. In addition, imatinib potently inhibits c-Kit and platelet derived growth factor receptors (PDGFRs), underlying its clinical use in the treatment of c-Kit-positive gastrointestinal stromal tumors (GIST) and PDGFR-associated myeloproliferative diseases. Interestingly, recent clinical reports have described improved glycemic control in patients with type 1 or type 2 diabetes taking imatinib for CML or chronic myeloproliferative disease [4]. Furthermore, preclinical studies have shown that imatinib treatment has efficacy in the non-obese diabetes (NOD) mouse model of T1D [5]. While several mechanistic studies suggest that inhibition of c-Abl and PDGFR are important for efficacy of imatinib in NOD diabetes [4], [5], the contribution of c-Kit inhibition to this activity has not been clearly addressed.

In this study, we engineer the T670I mutation into the mouse *c-kit* locus, an allele originally identified in GIST patients with refractory responses to imatinib [6], and bred the strain onto the NOD background to generate imatinib-resistant c-Kit mice that develop T1D. We characterize the imatinib response of diabetic NOD mice expressing wild-type or T670I mutant *c-kit* alleles and ask whether inhibition of c-Kit is required for efficacy of imatinib in this model. These results allow further definition of the target profile for tyrosine kinase inhibitor drug discovery programs focused on T1D treatment.

## Methods

### Generation of NOD.c-Kit^T670I^ mutant mice

A targeted mutation was introduced into exon 14 of the mouse *c-kit* allele to generate the T670I mutation (ACA→ATA). To track the presence of the knock-in allele, a *Bgl-II* restriction site (tgatct→agatct) was created by silent mutation 18 nucleotides 3′ of the T670I codon. A neomycin resistance gene flanked by *frt* sites served as a selectable marker after transfection of the targeting construct into Bruce 4 ES cells. G418-resistant ES cell clones were screened by PCR and confirmed by Southern blot using probes outside the homology arms. Three clones were injected into blastocysts from B6(Cg)-*Tyr^c-2J^*/J mice. Offspring from chimaeras of clone C9 that had transmitted the mutation through the germline were crossed with a FLP-deleter strain to remove the *frt*-flanked Neo-cassette. Neo-deleted offspring from this cross were bred to the NOD strain for 10 generations. Multiple known genomic insulin-dependent diabetes (Idd) loci (including CTLA4, IL2/ptpn8, MHC, CD25, insulin 1 and insulin 2) were analyzed. These loci were fixed from the NOD background strain before intercrossing mice heterozygous for the *c-Kit^T670I^* allele to generate cohorts for experiments.

All animal experiments were performed in strict accordance with the Genomics Institute of the Novartis Research Foundation Institution Animal Care and Use Committee (GNF IACUC) and Novartis Animal Welfare policies and guidelines, and under GNF IACUC approved protocol #11-291. Mice were housed in groups no greater than five in individually ventilated cages inside of climate controlled rooms on a twelve hour light/dark cycle. Animals showing signs of clinical deterioration or severe hyperglycemia confirmed by two consecutive blood glucose measurements greater than 600 mg/dl were euthanized according to the IACUC approved protocol using isofluorane inhalation followed by cervical dislocation (GNF PAR C-SOP TECH19).

### Confirmation of imatinib resistance

Hematopoietic stems cells (HSCs) were isolated from femurs of either NOD.c-Kit^T670I^ or wild-type NOD.c-Kit^wt^ littermate mice. HSCs were expanded in the presence or absence of 5 µM imatinib as previously described [7] for 6 days before being stained with rat anti-mouse CD117 (c-Kit)-APC (BD #553356) and anti-mouse Sca-1-PerCP Cy5.5 (eBioscience #45-5981-82) and analyzed by LSR II flow cytometer (Becton Dickinson).

### Monitoring for diabetes onset

Female NOD.c-Kit^T670I^ and NOD.c-Kit^wt^ littermates were monitored weekly for hyperglycemia from 6 weeks of age, using an Embrace Blood Glucose Monitoring System (Omnis Health) and a single drop of blood obtained from a small tail prick. Mice with blood glucose values (BGVs) of 200–400 mg/dl were re-screened the following day and enrolled in studies with a second BGV reading of 300–400 mg/dl. The first treatment dose was administered on the day of the confirmatory BGV test.

### Imatinib treatment

Recent onset diabetic NOD.c-Kit^T670I^ and NOD.c-Kit^wt^ mice were treated with either PBS or imatinib (Selleck Chemicals Ltd, cat.# S1026) prepared in distilled water and dosed by intra-peritoneal injection at 50 mg/kg/mouse, twice a day, for 21 consecutive days. Blood glucose values were assessed at a minimum frequency of 3 days/week, for three weeks during the course of treatment.

## Results

**Figure 1:**
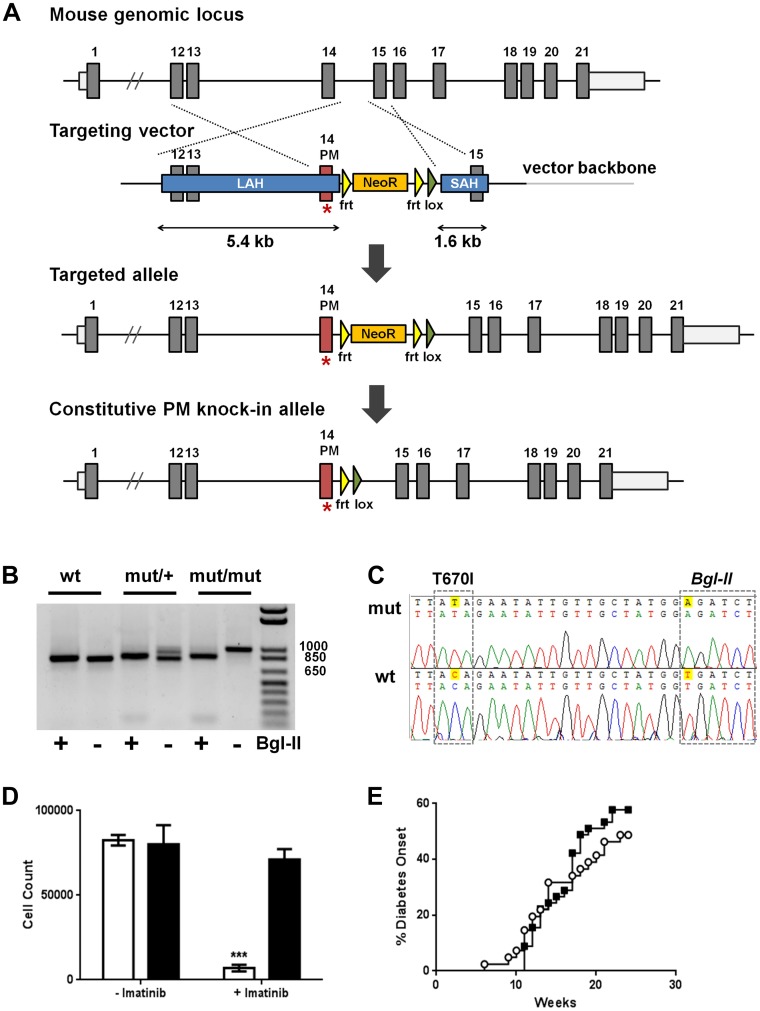
NOD.c-Kit^T670I^ mice are imatinib resistant and develop diabetes. A. Targeting strategy for generation of NOD.c-Kit^T670I^ mice. PCR genotyping (**B**) and sequence traces (**C**) of wild-type (wt) and mutant (mut) mice using primers F and R to generate fragments of 795 bp and 967 bp for wt and mut alleles, respectively. The T670I codon change and accompanying introduction of *Bgl-II* restriction site are highlighted. **D.** Expansion of c-Kit^+^/Sca-1^+^ murine HSCs from either NOD.c-Kit^T670I^ (black bar) or NOD.c-Kit^wt^ (white bar) littermates in the presence or absence of 5 µM imatinib. (*** p = 0.0002, as determined by two-way ANOVA). **E.** NOD.c-Kit^T670I^ (black square) mice develop diabetes comparably to NOD.c-Kit^wt^ (white circle) littermates (n = 41–45 mice/group; average age of diabetes onset = 14.5 weeks in NOD.c-Kit^wt^ mice and 15.5 weeks in NOD.c-Kit^T670I^ mice).

The T670I allele of *c-Kit* is a mutation originally identified in GIST patients with refractory responses to imatinib [6]. Like other threonine “gatekeeper” mutants identified in TKI resistance [6], [8]–[11], the exchange of threonine for a bulkier isoleucine sterically blocks imatinib from entering the ATP binding pocket, reduces inhibitor binding stability and affinity, and confers drug resistance [8]–[11]. To develop imatinib-resistant c-Kit NOD mice, we generated mice that carried the *c-kit* T670I mutation (Figure 1a–c), and then bred this allele onto the NOD background for 10 generations and until known genomic insulin-dependent diabetes (Idd) loci were fixed. Henceforth, mice homozygous for the T670I allele will be referred to as NOD.c-Kit^T670I^ mice, while control littermates homozygous for the wild-type allele will be referred to as NOD.c-Kit^wt^ mice.

To determine whether c-Kit activity in NOD.c-Kit^T670I^ mice was resistant to imatinib, we characterized the effect of imatinib on expansion of hematopoietic stem cells (HSCs), a process dependent on c-Kit [12]. We isolated HSCs from either NOD.c-Kit^T670I^ or NOD.c-Kit^wt^ littermate mice and expanded them *in vitro* in the presence or absence of 5 µM imatinib. After six days, we observed that expansion of HSCs from NOD.c-Kit^wt^ mice was substantially inhibited with addition of imatinib, as expected. In contrast, expansion of HSCs from NOD.c-Kit^T670I^ mice was not inhibited by imatinib, confirming that the *c-kit* allele of NOD.c-Kit^T670I^ mice is indeed resistant to imatinib (Figure 1d).

**Figure 2:**
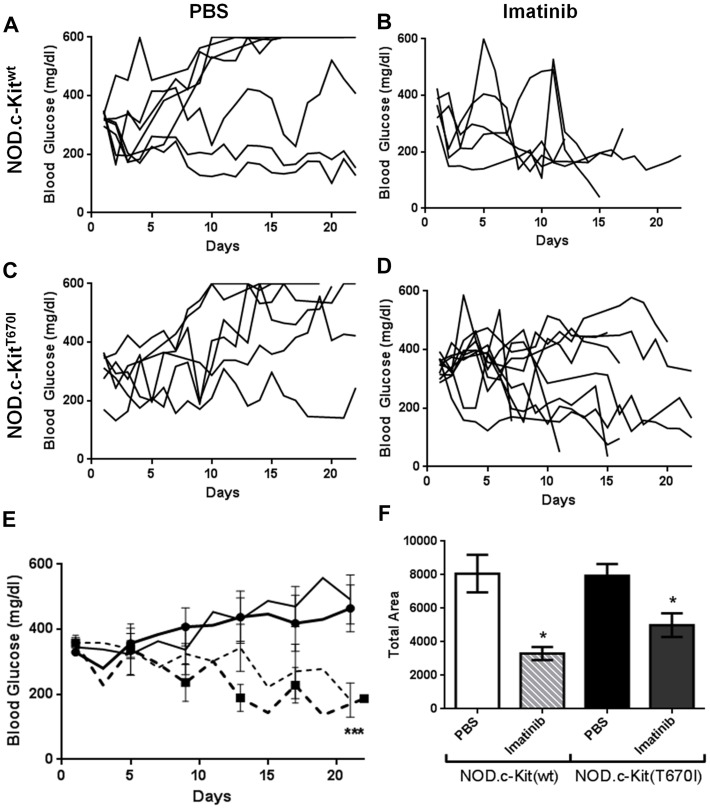
Diabetic NOD.c-Kit^T670I^ mice are sensitive to imatinib treatment. Blood glucose values for individual diabetic NOD.c-Kit^wt^ mice treated with either PBS (**A**) or imatinib (**B**), in comparison to diabetic NOD.c-Kit^T670I^ mice treated with either PBS (**C**) or imatinib (**D**) for 21 consecutive days. **E.** Average blood glucose values in diabetic NOD.c-Kit^wt^ or NOD.c-Kit^T670I^ mice treated with either PBS ((thick, solid line) NOD.c-Kit^wt^; (thin, solid line) NOD.c-Kit^T670I^) or imatinib ((thick, dashed line) NOD.c-Kit^wt^; (thin, dashed line) NOD.c-Kit^T670I^) for 21 consecutive days (n = 5–10 mice/group; *** p<0.0001 between PBS vs. imatinib within each mouse group for entire data set, as determined by one-way ANOVA). Note there was no statistically significant difference between NOD.c-Kit^wt^ and NOD.c-Kit^T670I^ within imatinib treatment groups. **F.** Area under curve for diabetes progression over 21 days comparing PBS ((open, white square) NOD.c-Kitwt; (closed, black square) NOD.c-Kit^T670I^) or imatinib ((grey, slashed square) NOD.c-Kit^wt^; (grey, closed square) NOD.c-Kit^T670I^) treated mice. (* p≤0.01 between PBS vs. imatinib within each mouse group, as determined by one-way ANOVA).

Subsequently, NOD.c-Kit^T670I^ and NOD.c-Kit^wt^ mice mice were monitored for diabetes onset. We found that both groups of mice developed diabetes with equivalent incidence and age of onset (Figure 1e). Recently diagnosed diabetic NOD.c-Kit^T670I^ and NOD.c-Kit^wt^ mice were dosed daily with imatinib over a period of three weeks, and blood glucose values (BGVs) were monitored at least every three days. We found that imatinib had comparable efficacy in reducing BGVs toward normal levels in both groups of mice (Figure 2 and Figure S1). Therefore, these data demonstrate that inhibition of c-Kit is not required for efficacy of imatinib in T1D.

## Discussion

Using a novel approach, we generated a genetically engineered mouse model of type 1diabetes that carries an imatinib-resistant c-Kit^T670I^ mutation and develops diabetes to define a target profile. Importantly, both groups of mice comparably develop diabetes and respond to imatinib treatment, ruling out any major contribution of c-kit inhibition in the mechanism of imatinib in this setting. Earlier studies by Louvet et. al. showed that neither an inhibitory anti-c-Kit antibody nor PLX647, a c-Kit/c-Fms inhibitor, had efficacy in diabetic NOD mice [5]. However, these studies were either complicated by anaphylaxis and death, or yielded weak, non-durable responses to inhibitor treatment leading the authors to conclude that c-Kit inhibition was not sufficient for efficacy. Our data provide genetic evidence consistent with Louvet et al, and further show that c-Kit inhibition is not required for efficacy by imatinib. Together, these studies indicate that the primary mechanism of action for imatinib efficacy in T1D is not through inhibition of c-Kit but probably through inhibition of other tyrosine kinase receptor targets such as c-Abl or PDGFR. Indeed, previous studies have implicated c-Abl and/or PDGFR inhibition by imatinib as likely targets in diabetes hyperglycemia reversal [5], [13].

Preclinical efficacy in NOD T1D has had poor positive predictive power in translation to clinical trials of human T1D. However, anecdotal reports from CML patients suggest that tyrosine kinase inhibitors may provide long-term benefit in reversing hyperglycemia in diabetic patients. As a targeted agent, imatinib is better tolerated than non-specific chemotherapeutics, and has been used in a pediatric population [3]. These findings have spurred efforts to initiate a controlled clinical trial evaluating the efficacy of imatinib in T1D patients [14]. However, the mechanism of action for imatinib and other tyrosine kinase inhibitors in treating mouse models of diabetes remains unclear. Studies that address this are crucial to future drug development in which improved, targeted therapies can be generated with better safety profiles. Our data support the development of inhibitors that do not target c-Kit, as inhibition of c-Kit is neither sufficient [5], nor is it required for efficacy of imatinib in NOD T1D. Importantly, loss of function alleles of *c-kit* have been adversely tied to impaired spermatogenesis, mast cell depletion, melanocyte dysfunction, and mild cytopenia [13]. Concerning treatments that may affect a pediatric population, tyrosine kinase therapies have also demonstrated an increased risk associated with impaired bone metabolism and growth retardation in prepubescent children [3], [15]. Notably, effects on bone have been attributed to inhibition of c-Kit signaling in osteoclasts [15]. In conclusion, this study provides important data in refining tyrosine kinase inhibitors to retain desired targets for the treatment of T1D while excluding irrelevant targets, minimizing potentially adverse events and improving the therapeutic index for new drug candidates.

## Supporting Information

Figure S1 **Blood glucose values at Day 1 vs. 21 of treatment.** Average blood glucose values in diabetic NOD.c-Kit^wt^ or NOD.c-Kit^T670I^ mice treated with either PBS or imatinib at day1(▪) versus day 21 (□) of treatment (n = 5–10 mice/group; * p<0.05 between day 1 vs. day 21 within treatment groups.)
(TIF)

## References

[1] Writing Group for the SfDiYSG (2007) Dabelea D, Bell RA, D'Agostino RB Jr, Imperatore G, et al (2007) Incidence of diabetes in youth in the United States. JAMA 297: 2716–2724. 17595272 10.1001/jama.297.24.2716

[2] Karvonen M, Viik-Kajander M, Moltchanova E, Libman I, LaPorte R, et al (2000) Incidence of childhood type 1 diabetes worldwide. Diabetes Mondiale (DiaMond) Project Group. Diabetes Care 23: 1516–1526. 11023146 10.2337/diacare.23.10.1516

[3] Suttorp M, Millot F (2010) Treatment of pediatric chronic myeloid leukemia in the year 2010: use of tyrosine kinase inhibitors and stem-cell transplantation. Hematology Am Soc Hematol Educ Program 2010: 368–376. 21239821 10.1182/asheducation-2010.1.368

[4] Welsh N (2012) Does the small tyrosine kinase inhibitor Imatinib mesylate counteract diabetes by affecting pancreatic islet amyloidosis and fibrosis? Expert Opin Investig Drugs 21: 1743–1750. 10.1517/13543784.2012.724398 22998750

[5] Louvet C, Szot GL, Lang J, Lee MR, Martinier N, et al (2008) Tyrosine kinase inhibitors reverse type 1 diabetes in nonobese diabetic mice. Proc Natl Acad Sci U S A 105: 18895–18900. 19015530 10.1073/pnas.0810246105 PMC2596241

[6] Tamborini E, Bonadiman L, Greco A, Albertini V, Negri T, et al (2004) A new mutation in the KIT ATP pocket causes acquired resistance to imatinib in a gastrointestinal stromal tumor patient. Gastroenterology 127: 294–299. 15236194 10.1053/j.gastro.2004.02.021

[7] Boitano AE, Wang J, Romeo R, Bouchez LC, Parker AE, et al (2010) Aryl hydrocarbon receptor antagonists promote the expansion of human hematopoietic stem cells. Science 329: 1345–1348. 20688981 10.1126/science.1191536 PMC3033342

[8] Pricl S, Fermeglia M, Ferrone M, Tamborini E (2005) T315I-mutated Bcr-Abl in chronic myeloid leukemia and imatinib: insights from a computational study. Mol Cancer Ther 8: 1167–74. 10.1158/1535-7163.MCT-05-0101 16093432

[9] Tamborini E, Pricl S, Negri T, Lagonigro MS, Miselli F, et al (2006) Oncogene 45: 6140–6. 10.1038/sj.onc.1209639 16751810

[10] Tutone M, Lauria A, Almerico AM (2011) Bioinformation 6: 296–8. 10.6026/007/97320630007296 PMC3280498 22355224

[11] Azam M, Seeliger MA, Gray NS, Kuriyan J, Daley GQ (2008) Nat Struct Mol Biol 10: 1109–18. 10.1038/nsmb.1486 PMC2575426 18794843

[12] Bernstein A, Chabot B, Dubreuil P, Reith A, Nocka K, et al (1990) The mouse W/c-Kit locus. Ciba Found Symp 148: 158–166 discussion 166–172. 1690623

[13] Hagerkvist R, Sandler S, Mokhtari D, Welsh N (2007) FASEB 21: 618–28. 10.1096/fj.06-6910com 17135364

[14] Gitelman SE BJ (2013) Imatinib Treatment in Recent Onset Type 1 Diabetes Mellitus. wwwclinicaltrialsgov NCT01781975.

[15] Vandyke K, Fitter S, Dewar AL, Hughes TP, Zannettino AC (2010) Dysregulation of bone remodeling by imatinib mesylate. Blood 115: 766–774. 19890095 10.1182/blood-2009-08-237404

