# Supplementary figures and images for: Inhibition of c-Kit Is Not Required for Reversal of Hyperglycemia by Imatinib in NOD Mice
Source: PLoS One. 2014 Jan 15;9(1):e84900. doi: 10.1371/journal.pone.0084900 (PMC3893161; doi:10.1371/journal.pone.0084900)

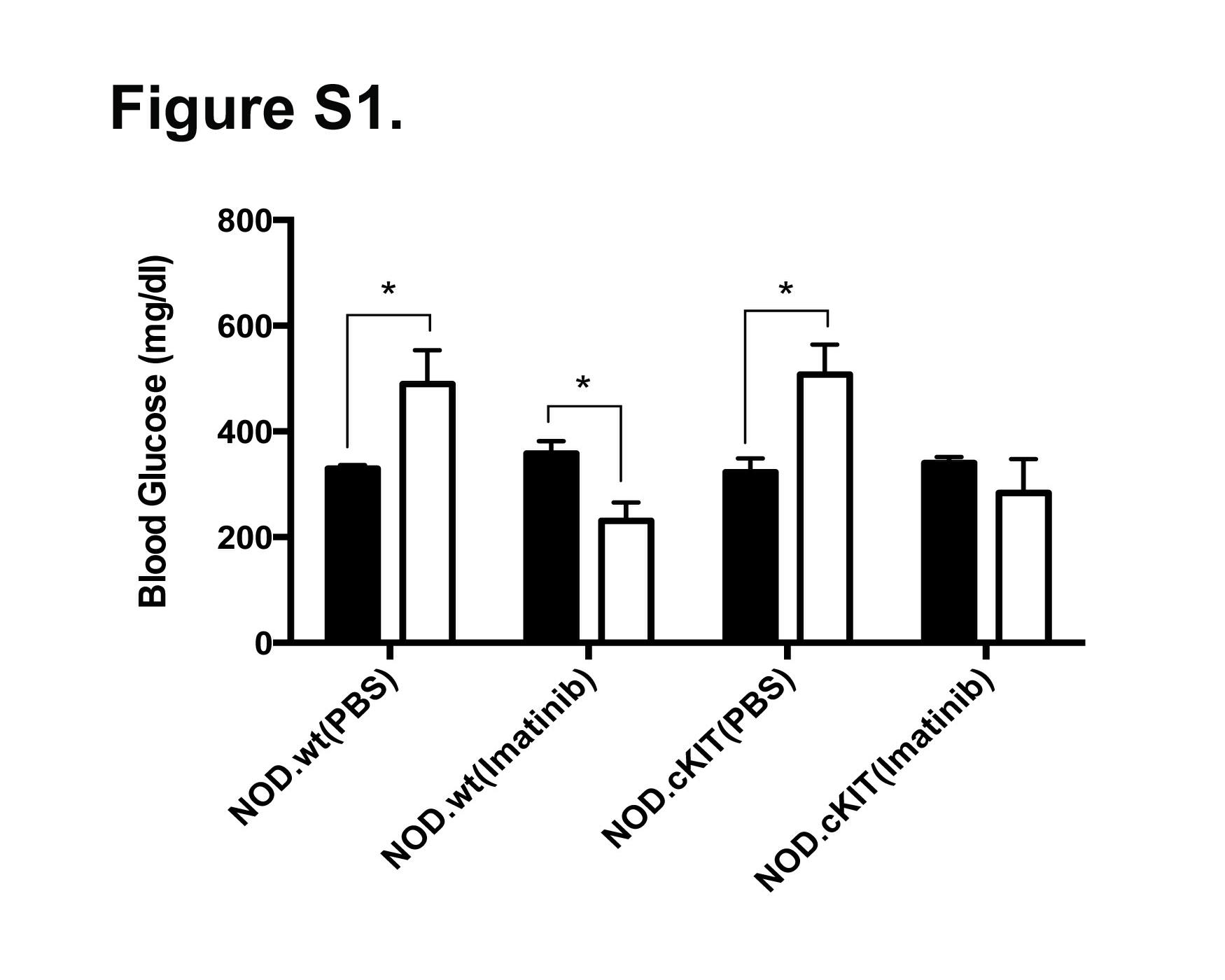

Supplement: Figure S1 — Blood glucose values at Day 1 vs. 21 of treatment. Average blood glucose values in diabetic NOD.c-Kitwt or NOD.c-KitT670I mice treated with either PBS or imatinib at day1(▪) versus day 21 (□) of treatment (n = 5–10 mice/group; * p<0.05 between day 1 vs. day 21 within treatment groups.) (TIF) [file pone.0084900.s001.tif]
